# Effects of Exercise Intensity on Spatial Memory Performance and Hippocampal Synaptic Plasticity in Transient Brain Ischemic Rats

**DOI:** 10.1371/journal.pone.0078163

**Published:** 2013-10-25

**Authors:** Pei-Cheng Shih, Yea-Ru Yang, Ray-Yau Wang

**Affiliations:** 1 Department of Physical Therapy and Assistive Technology, National Yang-Ming University, Taipei, Taiwan; 2 Department of Physical Therapy, Mackay Memorial Hospital, Taipei, Taiwan; National University of Singapore, Singapore

## Abstract

Memory impairment is commonly noted in stroke survivors, and can lead to delay of functional recovery. Exercise has been proved to improve memory in adult healthy subjects. Such beneficial effects are often suggested to relate to hippocampal synaptic plasticity, which is important for memory processing. Previous evidence showed that in normal rats, low intensity exercise can improve synaptic plasticity better than high intensity exercise. However, the effects of exercise intensities on hippocampal synaptic plasticity and spatial memory after brain ischemia remain unclear. In this study, we investigated such effects in brain ischemic rats. The middle cerebral artery occlusion (MCAO) procedure was used to induce brain ischemia. After the MCAO procedure, rats were randomly assigned to sedentary (Sed), low-intensity exercise (Low-Ex), or high-intensity exercise (High-Ex) group. Treadmill training began from the second day post MCAO procedure, 30 min/day for 14 consecutive days for the exercise groups. The Low-Ex group was trained at the speed of 8 m/min, while the High-Ex group at the speed of 20 m/min. The spatial memory, hippocampal brain-derived neurotrophic factor (BDNF), synapsin-I, postsynaptic density protein 95 (PSD-95), and dendritic structures were examined to document the effects. Serum corticosterone level was also quantified as stress marker. Our results showed the Low-Ex group, but not the High-Ex group, demonstrated better spatial memory performance than the Sed group. Dendritic complexity and the levels of BDNF and PSD-95 increased significantly only in the Low-Ex group as compared with the Sed group in bilateral hippocampus. Notably, increased level of corticosterone was found in the High-Ex group, implicating higher stress response. In conclusion, after brain ischemia, low intensity exercise may result in better synaptic plasticity and spatial memory performance than high intensity exercise; therefore, the intensity is suggested to be considered during exercise training.

## Introduction

Memory deficits are frequently noted after stroke, and are often related to impaired hippocampal function [1]. The hippocampus appears to be crucial for spatial memory and navigation. The spatial memory acquisition was also proved to be contributed to the induction of hippocampal long-term potentiation (LTP) [2]. Exercise has been suggested to be neuroprotective after stroke [3], [4]. Moreover, exercise was found to improve memory performance in transient global ischemic rat models [5]. The mechanisms underlying such beneficial effects on memory are often related to the hippocampal synaptic plasticity [6]. The brain-derived neurotrophic factor (BDNF) is one of the neurotrophin families, which is known not only to facilitate the neuronal survival and development, but also to modulate the synaptic plasticity [7]. In mammalian, BDNF is widely distributed in the central nervous system, especially in the hippocampus, which is thought to have contributions to learning and memory, particularly the spatial memory ability [8], [9]. BDNF plays an important role in the activity-dependent neuronal changes which are related to the memory acquisition and storage. For example, BDNF can enhance the synaptic transmission in the hippocampus by inducing local protein synthesis in the postsynaptic dendrites and presynaptic terminals [10], [11]. Running exercise has been proved to increase the expressions of hippocampal BDNF and synapsin-I, which contribute to the spatial memory performance in normal rats [12]. Dendritic morphological changes, such as increase dendritic aborization and dendritic spine density, have also been reported after physical exercise [13]. The enhanced synaptic protein expressions and postsynaptic structures can increase synaptic transmission and contribute to synaptic plasticity [14]. Interestingly, exercise intensity is noted to influence the hippocampal neurogenesis, BDNF and NMDA receptor mRNA expressions in normal rats, indicating the synaptic plasticity may be depended on the exercise intensity [15]. As the stress marker in rodents, corticosterone over-expressions were found to impair hippocampal BDNF mRNA transcription, indicating the increase of stress level can be harmful to hippocampal function [16]. However, the effects of exercise intensity on hippocampal synaptic plasticity and spatial memory after brain ischemia remain unclear.

In this study, we thus investigated the effects of exercise intensity on spatial memory performance and hippocampal synaptic plasticity, by demonstrating the expressions of hippocampal BDNF, Synapsin-I, PSD-95, and dendritic structures in brain ischemic rats.

## Methods

### Animals

Adult (8 week-old) Sprague-Dawley (SD) male rats were used. The rats were housed in pairs in a temperature-controlled (22±1°C) vivarium on a 12-hr light:dark cycle (lights on at 0700 hr) with free access to food and water. The Institutional Animal Care and Use Committee of the National Yang-Ming University approved all the experimental protocols.

### Focal Brain Ischemia: Middle Cerebral Artery Occlusion Model

Right middle cerebral artery occlusion (MCAO) was induced using standard microsurgical techniques as described previously [3]. In brief, the right middle cerebral artery trunk was ligated above the rhinal fissure with 10–0 suture thread with both common carotid arteries occluded with non-traumatic aneurysm clips for 1 hr. The complete interruption and reperfusion of blood flow was confirmed using an operating microscope.

### Exercise protocol

Forty-eight rats were subjected to this study. Before MCAO procedure, all rats experienced a 3-day familiarization of a motor-driven treadmill training (Treadmill Simplex II, Columbus Instruments, Ohio, USA) at the speed of 5 m/min, 5 min/day. After resting for 2 days, the MCAO procedure was performed, and the rats were randomly assigned to one of the three groups: sedentary (Sed), low-intensity exercise (Low-Ex) and high-intensity exercise (High-Ex) groups. Treadmill training was started at 24 hours post MCAO, 30 min per day, for a total of 14 consecutive days for the exercise groups. Rats in the Low-Ex group were trained on the treadmill at the speed of 8 m/min while rats in the High-Ex group at the speed of 20 m/min. Rats in the Sed group remained relatively inactive during the 14 days period.

### Morris Water Maze (MWM)

The Morris water maze test composes the acquisition phase and retention phase. In the acquisition phase, all rats in the three groups were trained on the MWM for 4 days (from day 10 to day 13 of the study period) with 3 training trials per day. In each trial, rats swam freely in the pool until they located the hidden platform, and those could not locate the platform within 90 s were gently guided to the platform. Escape latency (time needed to find the platform) was recorded during the trials. After completing the last treadmill training in the exercise groups or the comparable day of the Sed group (day 14), test for retention phase was executed, which the platform was removed from the pool. The rats were allowed to swim for 60 s during the retention test, and their behaviors were recorded. Two parameters were analyzed for comparisons during the retention phase: the duration of staying and the frequency of crossing the quadrant which the platform was previously located [17].

### Western blotting

Western blotting was used to identify the levels of BDNF, synapsin-I and PSD-95 protein expressions. All rats were sacrificed after the last trial of MWM test. Hippocampal samples were dissected out on ice, homogenized in the lysis buffer (137 mM NaCl, 20 mM Tris-HCl, 1% NP40, 10% glycerol, 1 mM PMSF, 10 µg/ml aprotinin, 1 µg/ml leupeptin), and centrifuged at 12500 rpm at 4°C for 30 min. For electrophoresis, equal amount of protein (10 µg) in each sample was separated in the 8% (for synapsin and PSD-95) or 12% (for BDNF) SDS-polyacrylamide gels, and then transferred onto the PVDF membranes. After blocking with the 3% BSA at room temperature for 1 hour and washed with 0.01% TBST buffer, the membranes were incubated with the anti-BDNF (1∶1000; Santa Cruz Biotechnology Inc., Santa Cruz, CA, USA), anti-synapsin-I (1∶3000; cell signaling, Beverly, MA, USA), anti-PSD-95 (1∶5000; cell signaling, Beverly, MA, USA) or anti-β-actin (1∶15000; Sigma-aldrich) antibodies at 4°C overnight. After washing with 0.01% TBST three times and incubated with the secondary antibodies at room temperature for 1 hour, the resulting signals were visualized with the enhanced chemiluminescence reagent (ECL; Millipore, Billerica, MA, USA). Optical densitometry was performed on each band using ImageJ software (National Institutes of Health, Bethesda, MD, USA). Protein content loaded was normalized by β-actin signal.

### Enzyme-linked immunosorbent assay (ELISA)

Serum corticosterone levels were detected using the corticosterone ELISA kit (Enzo Life Sciences, USA). Under deep anesthesia, blood samples were obtained by cardiac puncture. After centrifugation, serum samples were collected and diluted 10 times with the assay buffer provided by the kit. Competition ELISA procedures were conducted following the manufacturer's instructions and the sensitivity of the kit was approximately 27 pg/mL.

### Golgi stain procedure

We used golgi stain to identify the dendritic structure in rat hippocampus. After the last trial of MWM test, rats were anesthetized and sacrificed through transcardiac perfusion with ice-cold saline. The brain was immediately removed and placed in the mixture of mercuric chloride, potassium dichromate and potassium chromate solution provided by the FD Rapid Golgi Stain Kit (FD NeuroTechnologies, Inc., USA). Golgi stain procedures were following the manufacturer's instructions. The brain sections were cut with a cryostat, affixed to slides, dehydrated, cleared with Xylene, and coverslipped in Permount® (Merck, Darmstadt, Germany).

### Dendritic complexity analysis: Sholl analysis

**Figure 1:**
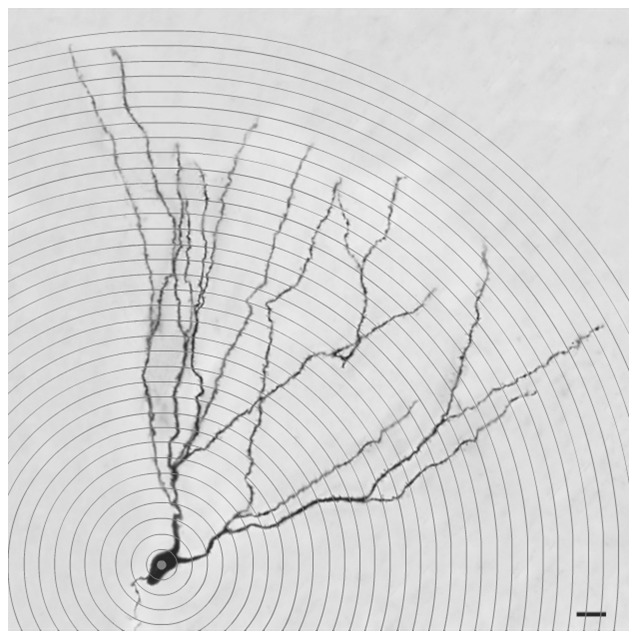
Sholl analysis of a dentate gyrus neuron. Scale bar: 20 µm.

To acquire images for analyzing the dendritic complexity, we scanned the hippocampal tissue using a 40X objective on an Olympus BX61 microscope. For dendritic complexity measurement, we used Sholl analysis [18] and sampled neurons from the hippocampal dentate gyrus (DG), CA1 and CA3 regions. Eight neurons from each region per animal were subjected to analysis. Analysis was performed by a customized Sholl analysis plug-in (Available at: http://www.biology.ucsd.edu/labs/ghosh/software/index.html) for ImageJ software (National Institutes of Health, Bethesda, MD, USA). As shown in Figure 1, concentric rings of increasing radius equivalent to 10 µm apart were used, and the numbers of intersections of the dendritic branches with each concentric ring were recorded [19].

### Dendritic spine density analysis

To acquire images for analyzing the spine density, we scanned the hippocampal dendritic segments using a 100X oil-immersion objective on an Olympus BX61 microscope. For quantification of the spine density, we sampled 8 neurons from each hippocampal DG, CA1 and CA3 regions. For each neuron, spine density was measured from eight 10 µm segments located on the distinct 2^nd^ or 3^rd^ dendritic terminal branches. Dendritic spine density was performed as number of spine/10 µm [20].

### Statistical analysis

The One-way ANOVA was used to compare the protein levels, dendritic spine density and data from the retention phase of MWM test among groups. The Two-way repeated measures ANOVA was used to compare the dendritic complexity and escape latency in the acquisition phase of MWM test among groups. The Tukey test was used as the post-hoc comparisons, and the significant level was set at p<0.05.

## Results

### Spatial memory performance- Morris water maze

**Figure 2:**
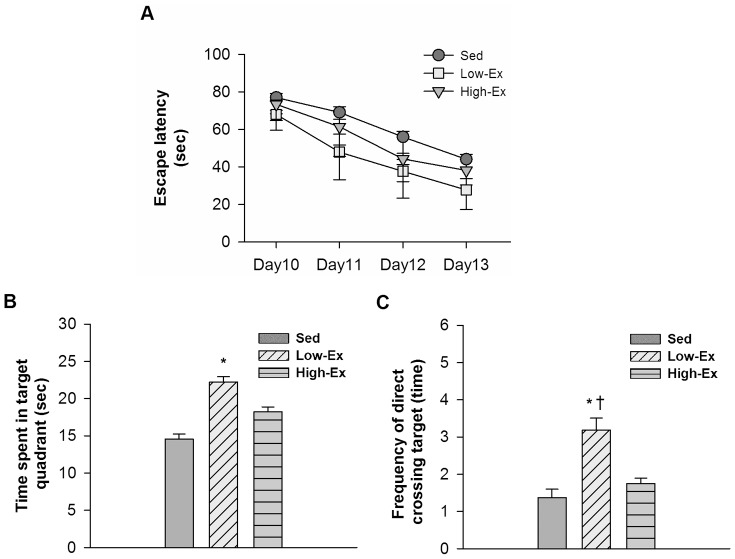
Spatial memory performance. (A) Escape latencies during acquisition phase - daily average of the 3 trials. (B) Time spent in the target quadrant during retention phase. (C) Frequency of direct crossing the target location. Data are all plotted as the mean+SEM). (*P<.05, **P<.01 versus Sed group; +P<.05 versus High-Ex group.).

Figure 2A demonstrates the daily mean escape latency of all groups during day10 to day13. The results of two-way repeated-measured ANOVA showed a significant within-subject time effect (P<.05), indicating all rats learned to perform the task during the acquisition phase. Post-hoc tests indicated rats in the Low-Ex group demonstrated shorter escape latency as compared with rats in the High-Ex (p<.05) and Sed (p<.01) groups on day11 to day13. Figure 2B shows the time spent in the training quadrant during the retention phase. Group differences were observed (p<.05) and post-hoc test revealed rats in the Low-Ex group spent longer time in the training quadrant compared with the Sed group (P<.05). For frequency of direct crossing the platform (Figure 2C), rats in the Low-Ex group also crossed more frequently in the training quadrant as compared with the other two groups (P<.05 versus High-Ex group; p<.01 versus Sed group).

### Hippocampal protein expression

**Figure 3:**
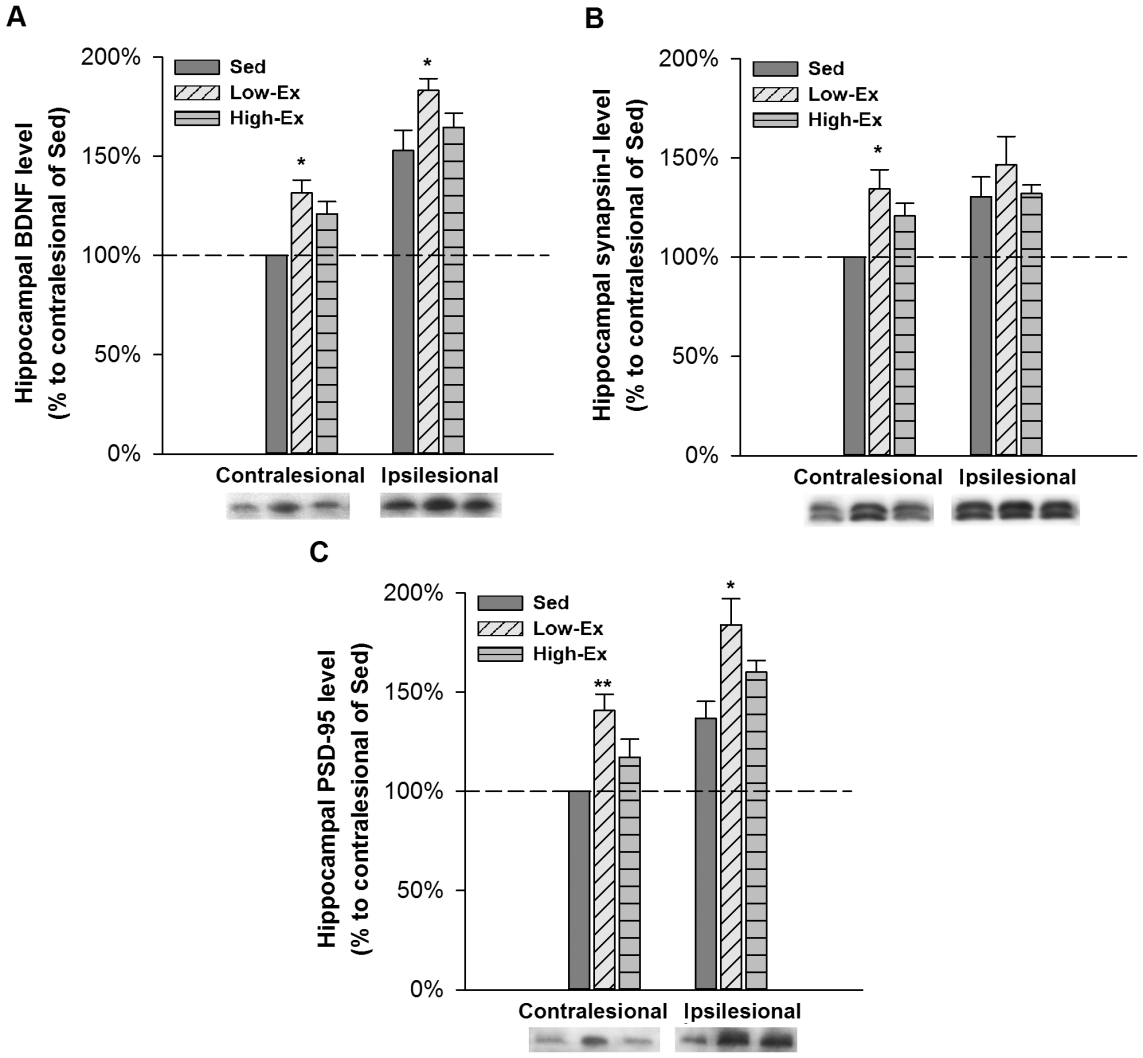
Protein expression in the hippocampus. (A) BDNF. (B) Synapsin-I. (C) PSD-95. Results are presented as percentage to the contralesional side of the Sed group. Data are all plotted as the mean±SEM. *P<.05,**P<.01versus Sed group.

Results of hippocampal protein levels by western blotting are presented as percentage to the contralesional side of the Sed group (Figure 3A–3C). We found that low-intensity exercise, not the high-intensity exercise, significantly increased the levels of BDNF (p<.05), synapsin-I (p<.05) and PSD-95 (p<.01) in the contralesional hippocampus as compared with the Sed group. Low-intensity exercise also significantly increased the levels of BDNF (p<.05) and PSD-95 (p<.05) in ipsilesional hippocampus as compared with the Sed group, whereas the high intensity exercise did not result in such effects.

### Hippocampal dendritic structure

**Figure 4:**
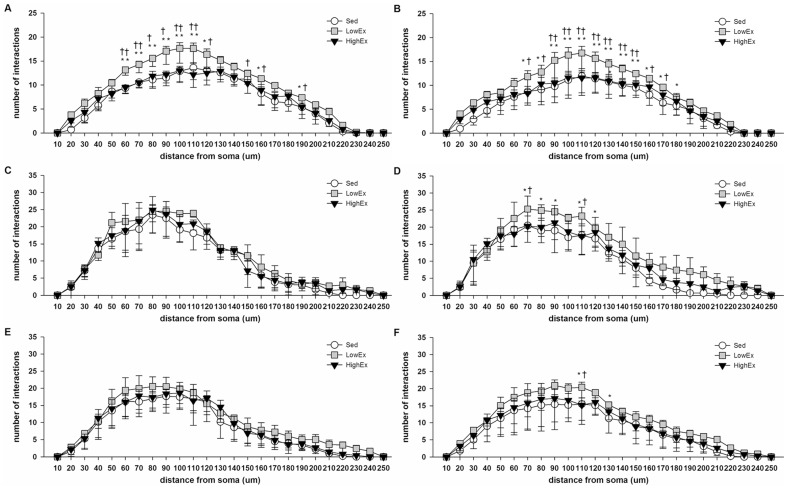
Hippocampal dendritic complexity. Dendritic complexity of: (A) Ipsilational hippocampal dentate gyrus. (B) Contralesional hippocampal dentate gyrus. (C) Ipsilational hippocampal CA1. (D) Contralesional hippocampal CA1. (E) Ipsilational hippocampal CA3. (F) Contralesional hippocampal CA3. Data are all plotted as the mean±SEM. *P<.05, **P<.01 versus Sed group; +P<.05, ++P<.01versus High-Ex group.

Two-way repeated ANOVA revealed a significant group*ring interaction in the DG of both hemispheres (p<.01). Post hoc test showed that rats in the Low-Ex group demonstrated an increase in the numbers of dendrite-ring intersection between 70 µm and 120 µm from the cell body as compared with the other two groups (Figure 4A–4B). In the CA1 and CA3 regions, group*ring interaction was only found in the contralesional hemisphere (p<.05 for both CA1 and CA3). Post hoc test further indicated the number of dendrite-ring intersection was significantly more in the Low-Ex group as compared with the High-Ex and Sed group (CA1: p<.01 compared with High-Ex and Sed group; CA3: p<.01 compared with Sed group, p<.05 compared with High-Ex group) (Figure 4C–4F).

**Figure 5:**
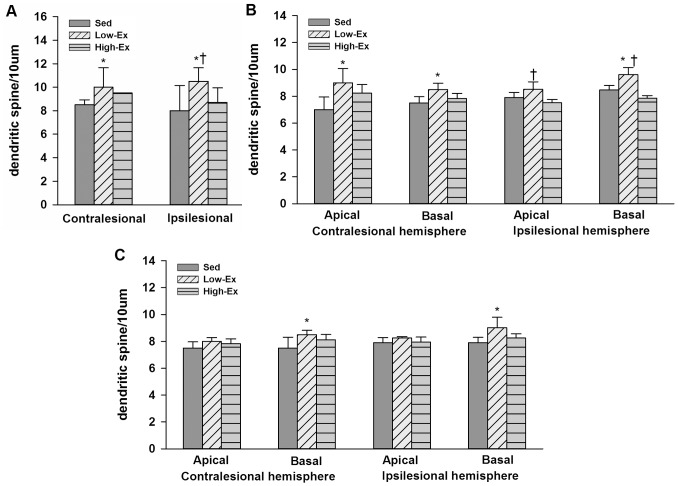
Hippocampal dendritic spine density. Dendritic spine density of: (A) Hippocampal dentate gyrus. (B) Hippocampal CA1. (C) Hippocampal CA3. Data are all plotted as the mean±SEM. *P<.05, **P<.01 versus Sed group; +P<.05 versus High-Ex group.

Rats in the Low-Ex group demonstrated more dendritic spines per 10 µm segment in the hippocampal DG and CA1 region of both hemispheres when compared with the Sed group (P<.05; Figure 5A–5B). Moreover, in the ipsilesional hippocampus, we observed that the dendritic spine density in the DG and CA1 region of the Low-Ex group were higher than the High-Ex group (p<.05). Low-intensity exercise also increased the dendritic spine density in bilateral hippocampal CA3 regions as compared with the Sed group (p<.05) (Figure 5C).

### Serum corticosterone concentration

**Figure 6:**
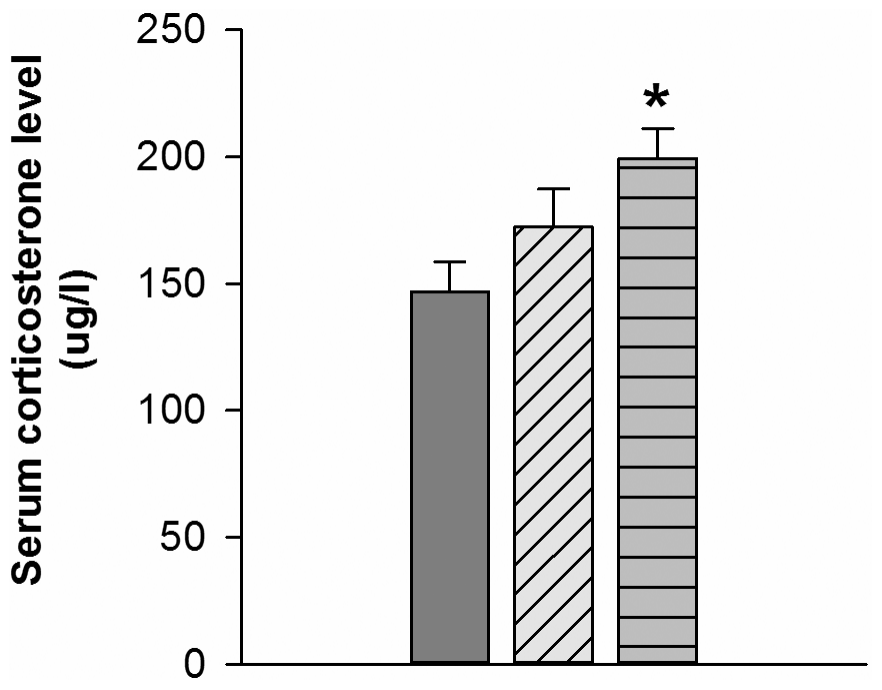
Level of serum corticosterone concentration. Data are all plotted as the mean±SEM. (*P<.05 versus Sed group.).

Figure 6 illustrates the serum corticosterone concentration among groups. Rats in the High-Ex group demonstrated higher serum corticosterone concentration than rats in the Sed group (P<.05). However, the corticosterone concentration was not significantly different between the Low-Ex and Sed group (P = 0.365).

## Discussion

The main findings of this study are that low-intensity, but not high-intensity, can improve spatial memory performance after brain ischemia. Such memory improvement is indicative of the structural and functional hippocampal plastic changes in bilateral hemispheres, including hippocampal BDNF, PSD-95 and dendritic complexity. In addition, high-intensity exercise does not exert such positive effects were probably due to the induced stress response.

The beneficial effects of low-intensity exercise have also been noted in other study. Shimida et al. found that after 4 weeks of low-intensity (8 m/min) treadmill training, the performance in object recognition task is significantly better than high-intensity (22 m/min) treadmill training in MCAO rats, and this improvement paralleled with the increase of neurons in the hippocampal DG [21]. In our study, we further demonstrated that after low-intensity treadmill training, there were structural changes, such as increase of dendritic complexity and dendritic spine density in the hippocampal DG, CA1 and CA3. Taking together, the low-intensity treadmill training may not only enhance the neurogenesis, but also promote the synaptogenesis as indicated in the present study.

Dendritic morphological changes were observed after physical and environmental experiences in other studies. For instance, environmental enrichment can enhance the hippocampal dendritic structural plasticity, including higher dendritic spine density and thicker PSD region, and thus contribute to spatial memory performance [22], [23]. The increase of dendritic spine density was also noted after wheel running in the diabetic mice [20]. In our study, a moderate correlation between the hippocampal dendritic structure and spatial memory performance was noted (r = .643, P< .01). It is not surprising to note such correlation, since most dendritic spines are located on the hippocampal excitatory synapse, and higher spine density contributes to the increase of excitatory neurotransmission [24]. To our knowledge, the present study is the first to investigate the effects of exercise intensity on the hippocampal dendritic morphology after brain ischemia. Our present results revealed that low intensity exercise can enhance the dendritic complexity and dendritic spine growth in all of the three subregions we investigated, the DG, CA1 and CA3 region. However, the dendritic changes in DG are most significant as compared with the other two regions. DG is a plastic region of the hippocampus where neurogenesis occurred, and exercise-induced LTP in DG has been found to accompany with the increasing dendritic complexity, spine density and neuronal progenitor in normal rats [25]. Dendritic damage and degeneration in the hippocampal DG are related to synaptic loss and impaired hippocampal function, since DG is the gate of hippocampus to receive excitatory inputs from the entorhinal cortex, and let information transmit into the hippocampus trisynaptic loop [26]. On the other hand, the neuronal activities in CA3 region provide information about current object location through the inputs from entorhinal cortex. The apical dendrites of CA1 neurons received CA3 output with afferent input from entorhinal cortex. Lesions in CA1 region may result in less spatial exploration behavior, suggesting its important role in this behavior [27], [28]. Based on above findings, the neuronal plastic changes in DG, CA1 and CA3 may all contribute to the improved performance in Morris water maze after low intensity exercise as demonstrated in our study.

In addition to structural plasticity induced by low intensity exercise, the functional aspects of synapse may also be enhanced as supported indirectly by the increase of BDNF levels. It is known that the existence of BDNF not only enhances synaptic response to tetanus stimulation and facilitates transient long-term potentiation (LTP), but also regulates actin motor complex to maintain LTP expression [29].

For synaptic protein concentrations, we observed a similar trend as in the dendritic structure; that is, only low-intensity exercise enhanced the expressions of hippocampal BDNF and PSD-95 in bilateral hemispheres, and synapsin-I in the contralesional hemisphere. The levels of PSD-95 and synapsin-I are possibly regulated by the BDNF, since there are several studies reporting the dominant role of BDNF in the hippocampal synaptic plasticity. Inhibition of BDNF-TrkB signalings blocked the exercise-induced BDNF, synapsin-I and CREB expressions, and abolished the improvement of spatial memory [10], [12]. Synapsin-I is a terminal specific phosphoprotein that can regulate the trafficking of small synaptic vesicles within the presynaptic terminal [30]. Blockade of BDNF signaling was also found to impair the hippocampal PSD-95 expression and decrease the dendritic growth [31]. The BDNF can enhance the transmission and transportation of PSD-95 from the soma to the dendritic terminal through the PI3K-AKT pathway and bind to the postsynaptic molecules, which can regulate dendritic aborization and dendritic growth [32]. Furthermore, BDNF-TrkB signaling may also play an important role in facilitating glutamate receptor stabilization through the PSD-95 for excitatory synapse formation. In addition, overexpression of PSD-95 was contributed to increase spine numbers, size and synaptic efficacy [14]. These protein changes may all contribute to the hippocampal synaptic plasticity and spatial memory as noted in our study.

Previous study showed that low intensity exercise resulted in more positive effects on improving synaptic plasticity than high intensity exercise in normal rats. [15]. In the present study, we further demonstrated such intensity-related beneficial effects in brain ischemic rats may be due to the induced stress levels. Stress has been found to affect the learning and memory process by reducing the expression of hippocampal BDNF, dendritic arbors and dendritic spine density [33], [34]. Our study observed that the serum corticosterone level was significantly increased in the high-intensity exercise group. Corticosterone is known to interact negatively with BDNF mRNA and down-regulate BDNF signaling in the hippocampus. The possible mechanisms of corticosterone to affect BDNF expression were often suggested to relate to the glucocorticoid receptor (GR) in hippocampus. Numakawa and his colleagues noted the interaction between steroid hormones and neurotrophin BDNF are mainly mediated by GR. They found the GR interacts with TrkB receptor and affects glutamate release stimulated by BDNF signaling pathway. The enhancement of TrkB-GR interaction can activate BDNF-PLCγ signaling and improves neurotransmitter release, while corticosterone plays a negative role in TrkB-GR interaction. Corticosterone exposure reduced TrkB-GR interaction, so as to result in dysfunction of BDNF and decrease in BDNF-mediated glutamate release. [35]. Furthermore, Chronic corticosterone exposure can lead to reduce the TrkB-GR interaction and disrupt the BDNF mRNA transcription, and thus decrease BDNF protein level [16]. Subcutaneous corticosterone injection was also found to decrease the hippocampal BDNF mRNA level, especially in the DG and CA1 region with a dose-dependent effect [36]. Chronic stress was also reported to suppress the dendritic complexity, especially in the hippocampal CA1 and CA3 [37], [38]. In addition, dendritic spine density loss was found in the apical dendrites of CA3 and basal dendrites of CA1 after corticotrophin releasing [39], [40]. Therefore, the insignificant effects on memory of high intensity exercise noted in our study may be at least partly due to the high level of corticosterone.

Previously, we demonstrated high-intensity treadmill training for 2 weeks resulted in better motor performance and decreased in infarct volume than low-intensity training in brain ischemic rats [41]. Our present and previous results may indicate different effects of exercise intensity on the motor and cognitive functions, which may be due to the decrease of BDNF specifically in the hippocampus after the corticosterone exposure [16]. Therefore, the high-intensity training may exert beneficial effects on the motor function, but not equally on the cognitive function.

There are some limitations of this study should be noted. First, we only examined the levels of BDNF, synapsin-I and PSD-95 in whole hippocampal tissues, therefore, we cannot elucidate these changes with dendritic modifications in specific hippocampal subregions. Second, we only measured the corticosterone levels in serum, but not the hippocampus; therefore, the dendritic structural changes in DG, CA1 and CA3 influenced by corticosterone are only supported indirectly.

## Conclusions

Our study showed that after brain ischemia, low intensity exercise resulted in better effects on hippocampal synaptic plasticity and spatial memory, whereas high intensity exercise did not exert such beneficial effects possibly due to the stress induced by high intensity exercise.
